# Self-Assembled Formation of Well-Aligned Cu-Te Nano-Rods on Heavily Cu-Doped ZnTe Thin Films

**DOI:** 10.1186/s11671-016-1741-x

**Published:** 2016-11-29

**Authors:** Jing Liang, Man Kit Cheng, Ying Hoi Lai, Guanglu Wei, Sean Derman Yang, Gan Wang, Sut Kam Ho, Kam Weng Tam, Iam Keong Sou

**Affiliations:** 1 Department of Physics and William Mong Institute of Nano Science and Technology, The Hong Kong University of Science and Technology, Room 4459, Academic Building, Clear Water Bay, Kowloon, Hong Kong People’s Republic of China; 2 Department of Physics, South University of Science and Technology of China, 1088 Xueyuan Rd., Nanshan District, Shenzhen, Guangdong People’s Republic of China; 3 Faculty of Science and Technology, University of Macau, E11 Avenida da Universidade, Taipa, Macau, China

**Keywords:** MBE, Heavily Cu-doped ZnTe, Surface modulation, Self-assembled nano-rods

## Abstract

Cu doping of ZnTe, which is an important semiconductor for various optoelectronic applications, has been successfully achieved previously by several techniques. However, besides its electrical transport characteristics, other physical and chemical properties of heavily Cu-doped ZnTe have not been reported. We found an interesting self-assembled formation of crystalline well-aligned Cu-Te nano-rods near the surface of heavily Cu-doped ZnTe thin films grown via the molecular beam epitaxy technique. A phenomenological growth model is presented based on the observed crystallographic morphology and measured chemical composition of the nano-rods using various imaging and chemical analysis techniques. When substitutional doping reaches its limit, the extra Cu atoms favor an up-migration toward the surface, leading to a one-dimensional surface modulation and formation of Cu-Te nano-rods, which explain unusual observations on the reflection high energy electron diffraction patterns and apparent resistivity of these thin films. This study provides an insight into some unexpected chemical reactions involved in the heavily Cu-doped ZnTe thin films, which may be applied to other material systems that contain a dopant having strong reactivity with the host matrix.

**Electronic supplementary material:**

The online version of this article (doi:10.1186/s11671-016-1741-x) contains supplementary material, which is available to authorized users.

## Background

ZnTe bulk crystal has increasing importance for various advanced semiconductor applications such as solar cells, blue-green laser diodes, terahertz imaging, electro-optic detector, and holographic interferometry [1–7]. The success of doping control of ZnTe thin films also makes ZnTe a suitable candidate for various optoelectronic device materials [8, 9]. ZnTe is well known to exhibit a pronounced preference for p-type due to self-compensation via Zn vacancies [10, 11]. Among many options for p-type doping of ZnTe, nitrogen (N) doping [12–16] and copper (Cu) doping [17, 18] have been mostly studied. Even though N doping using a radiofrequency (RF) plasma source has been proved to offer better doping characteristics, Cu doping has also been widely accepted as a good candidate as it can be achieved using various inexpensive ways including chemical immersions, sputtering, electrochemical deposition, and thermal evaporation [19–23]. Among previous reports on Cu doping of ZnTe thin films, most of them mainly focus on their electrical properties as a function of the Cu concentration without performing their structural and chemical analysis. Akkad and Abdulraheem recently provided evidence for the formation of the ternary zinc-copper-telluride alloy films containing Cu concentration above ~4 at.% prepared using RF magnetron sputtering [24], indicating that a structural or phase change could occur as Cu concentration in ZnTe reaches the over-doped regime. In this study, we discovered the formation of one-dimensional (1D) surface modulation and Cu-Te nano-rods for heavily Cu-doped ZnTe thin films grown by MBE. These findings explain the unusual observations in reflection high energy electron diffraction (RHEED) patterns and apparent resistivity of these thin films upon the increase of Cu cell temperature. Our work demonstrates that a highly reactive dopant could lead to formation of nanostructures in the host matrix, which may generally be applied to the studies of heavy doping using other physical or/and chemical vapor deposition approaches.

## Methods

In this work, ZnTe:Cu thin films were prepared in a VG V80H MBE system, which is equipped with in-situ RHEED to provide a real time monitoring of the thin film growth. Prior to the growth of these doped thin films, the growth of undoped ZnTe thin films on semi-insulating GaAs (001) substrates was firstly optimized by tuning the temperatures of both the Zn and Te effusion cells as well as the substrate. Growth temperatures were optimized at T_sub_ = 358 °C, T_Zn_ = 230 °C, and T_Te_ = 283 °C. Using these optimized temperatures, a set of 20 ZnTe:Cu samples with thicknesses around 850 nm were fabricated with the Cu cell temperature ranging from 840 to 1030 °C with a step of 10 °C. The room temperature resistivity measurements were carried out using a HL5500PC (Bio-Rad) system with needle probes contacting the indium electrodes made onto the samples in Van der Pauw geometry. Atomic force microscopy (AFM) characterizations were conducted by a Dimension 3100 AFM with a NanoScope IIIa controller (Digital Instruments) using tapping mode. Cross-sectional and plan-view high resolution transmission electron microscope (HRTEM) images were obtained by a JEOL 2010F TEM. The high resolution x-ray diffraction (HRXRD) measurements were performed with the PANalytical Multipurpose X-Ray Diffractometer. Time-of-flight secondary ion mass spectrometer (TOF-SIMS) was carried out using a PHI7200 SIMS system with Cs as the primary ion source.

## Results and Discussion

**Fig. 1:**
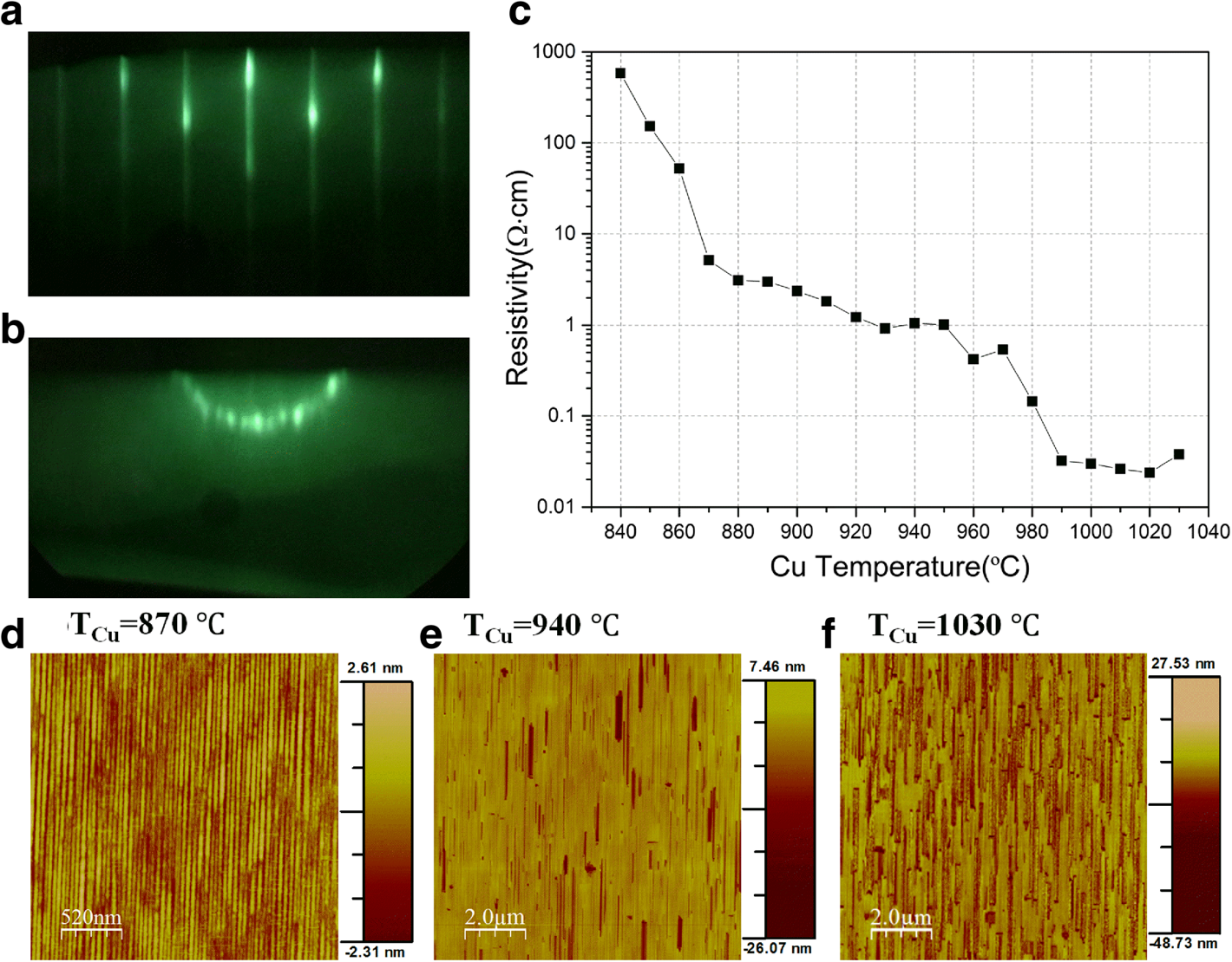
RHEED patterns of sample grown using T_Cu_ = 880 °C when e-beam aligned with **a** $\left[1\overline{1}0\right]$ and **b** [110] direction. **c** Apparent resistivity as a function of Cu cell temperature. **d**–**f** AFM images of three samples grown under Cu cell temperature at 870, 940, and 1030 °C

For T_Cu_ ≤ 860 °C, the observed RHEED patterns were similar to those of an optimized ZnTe thin film. Spotty patterns in the initial growth period were observed due to the large lattice mismatch between ZnTe and GaAs, and then they evolved into streaky and bright patterns. Starting from T_Cu_ ≥ 870 °C, streaky pattern as shown in Fig. 1a was observed when the $\left[1\overline{1}0\right]$ direction of the sample was aligned with the e-beam. While the sample was rotated, these straight streaks started to bend and the centered streak then evolved into a half circle as seen in Fig. 1b at a rotation angle of 90°, that is when the [110] direction of the sample was aligned with the e-beam. For the further 90°–180° rotation, a reverse order of bending with opposite bending direction proceeded. Such unusual RHEED patterns were firstly reported by us for an annealed Fe/ZnSe bilayer [25], which illustrates that these patterns came from a surface with one-dimensional surface modulation with the lateral scale around a few nanometers along the $\left[1\overline{1}0\right]$ direction due to the interaction between the Fe atoms and the ZnSe surface lattice. Thus, the similar patterns observed for the ZnTe:Cu thin films grown using T_Cu_ ≥ 870 °C is likely due to a similar interaction between Cu atoms and ZnTe surface lattice when the incorporated Cu atoms reach a certain concentration. It was further observed that such RHEED patterns got dimmer as T_Cu_ increases, though the overall patterns remain unchanged.

On the other hand, an unusual characteristic was also observed in the apparent resistivity of the as-grown ZnTe:Cu samples. Figure 1c displays the measured apparent resistivity of all 20 ZnTe:Cu samples, in which one can see that the apparent resistivity falls rapidly till T_Cu_ reaches 870 °C followed by a relatively slower decline as T_Cu_ further increases. For samples with T_Cu_ ≥ 990 °C, a characteristic sharp fall followed by a plateau region is observed. In the following paragraphs, we will describe various studies performed on the as-grown ZnTe:Cu thin films to understand the unusual characteristics on their RHEED patterns and apparent resistivity.

AFM surface imaging was conducted on a number of ZnTe:Cu thin films to reveal the underlying cause of their unusual RHEED patterns. The AFM images for samples grown using T_Cu_ ≤ 860 °C are similar to that observed on an ordinary MBE-grown thin film. As can be seen in Fig. 1d, the surface of the sample grown using T_Cu_ = 870 °C presents a [110] oriented 1D feature with non-uniform spacing around tens of nanometers, which is induced by the interaction between Cu atoms and the ZnTe surface similar to what we reported for the interaction between Fe atoms and the ZnSe surface [25], leading to the unusual RHEED pattern observed in samples grown using T_Cu_ ≥ 870 °C. Additionally, inspection of Fig. 1e, f reveals that besides the 1D feature with non-uniform spacing, some [110] oriented dented nano-trenches with width from a few tens up to a few hundreds of nanometers appears at samples grown using T_Cu_ ≥ 880 °C and their densities increase as T_Cu_ further increases. These nano-trenches explain why the unusual RHEED patterns got dimmer as T_Cu_ further increases from 870 °C since the addition of the nano-trenches will reduce the effective area of the background 1D feature.

**Fig. 2:**
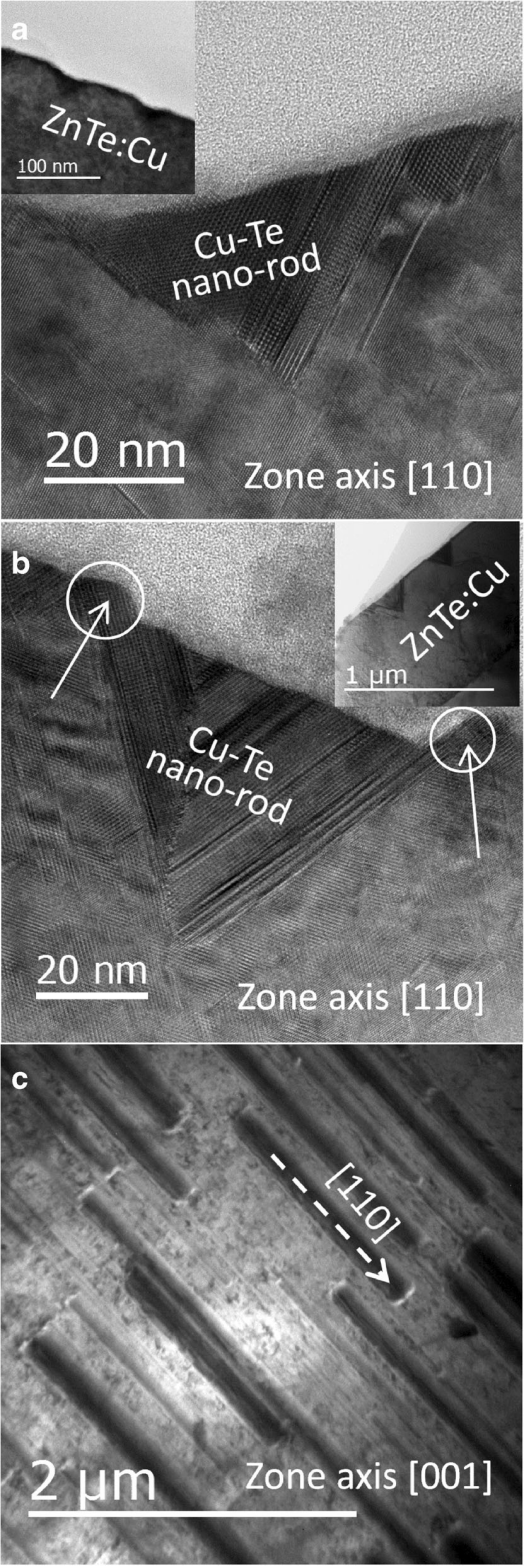
Cross-sectional TEM images of samples grown using Cu cell temperature at **a** 940 °C and **b** 1030 °C. *Insets* show the corresponding low-magnification images. **c** Plan-view TEM image of sample grown using T_Cu_ = 1030 °C in which some nano-rods near the surface can be clearly seen

In order to have a better understanding on the self-assembled mechanism and the chemical composition of the observed nano-trenches, cross-sectional TEM imaging together with energy-dispersive X-ray spectroscopy (EDS) were conducted on two samples grown using T_Cu_ = 940 and 1030 °C. Figure 2a, b displays the corresponding cross-sectional TEM images of an individual nano-trench in each of these two samples taken with a zone axis along the [110] direction. These images reveal that surprisingly, the nano-trenches have a triangular cross-sectional shape and from now on they are called nano-rods. The insets in Fig. 2a, b show the corresponding cross-sectional view with a lower magnification, indicating that the observed nano-rods exist only near the surface. Based on their cross-sectional TEM and AFM images, one can conclude that the nano-rods grow larger and denser as T_Cu_ increases. A plan-view TEM image of the sample grown using T_Cu_ = 1030 °C is displayed in Fig. 2c, which provides further evidence of the morphology of the aligned Cu-Te nano-rods. The results of EDS (see Additional file 1) performed on regions inside and outside the nano-rods indicate that the nano-rods are composed of mainly Cu-Te alloy with very rich Cu composition, roughly reaching a Cu:Te ratio close to 2:1.

**Fig. 3:**
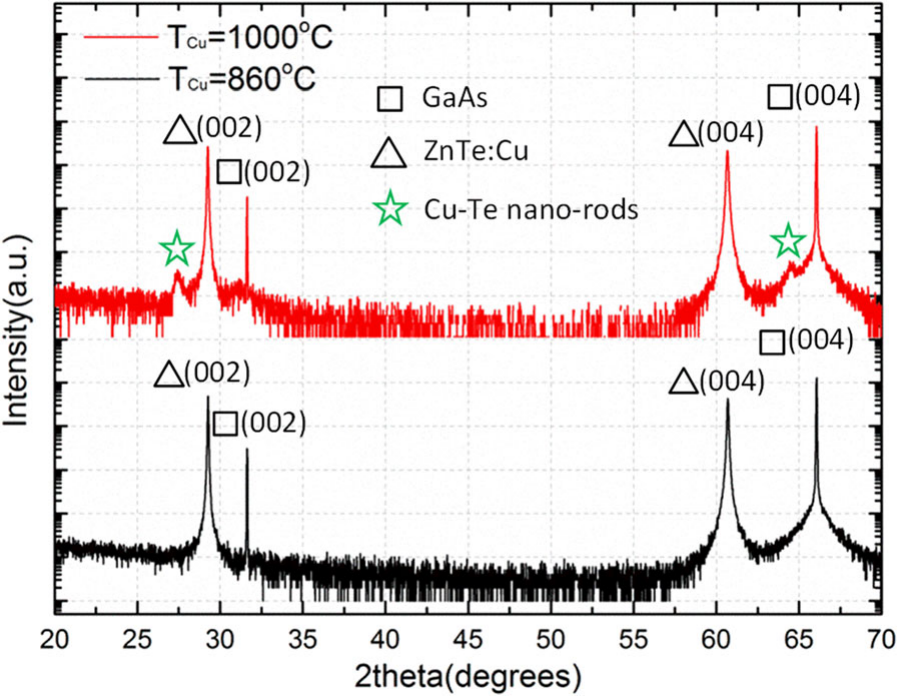
HRXRD results of samples grown using Cu cell temperature at 860 and 1000 °C

**Fig. 4:**
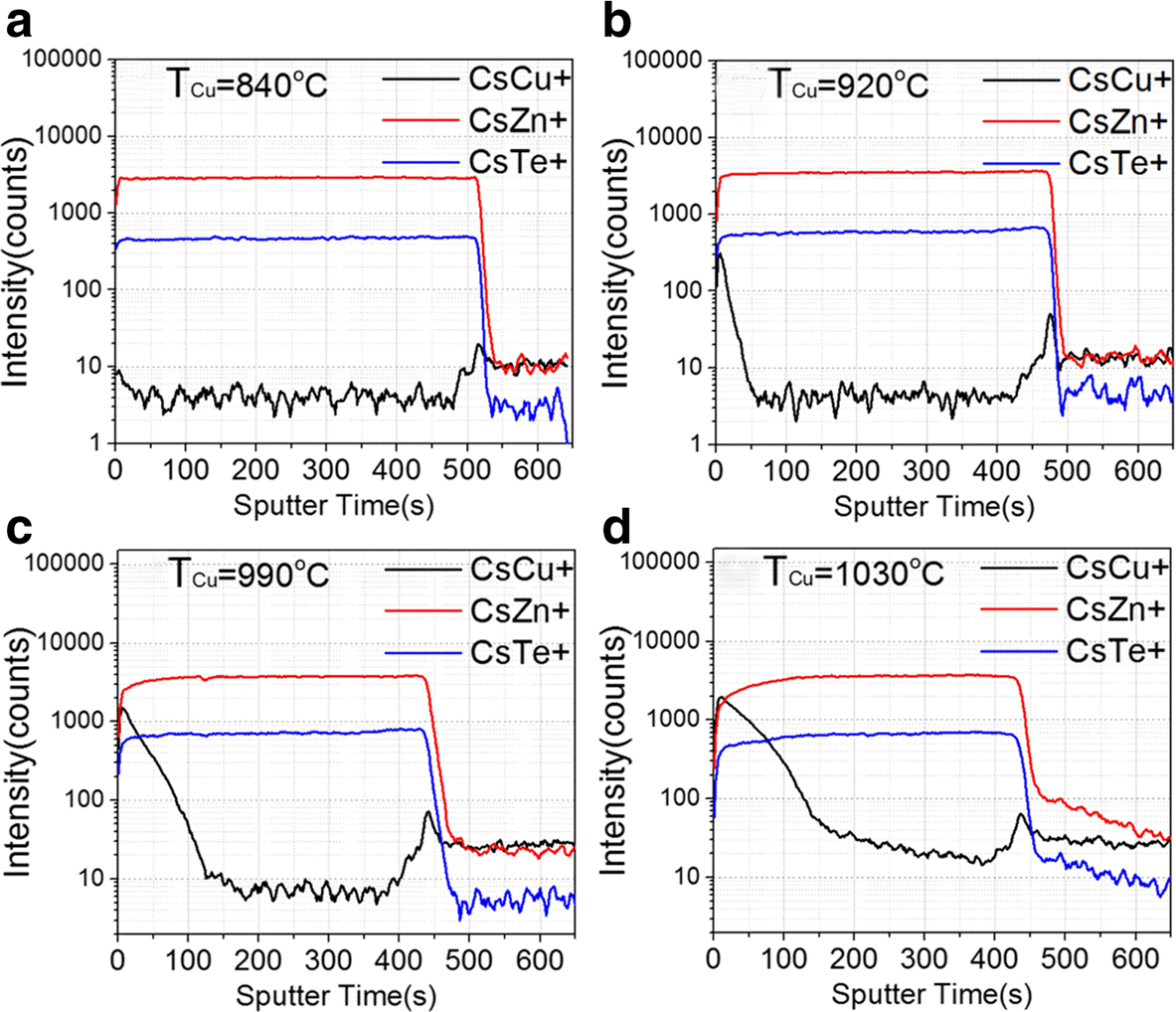
SIMS profiles of four ZnTe:Cu thin films using different Cu cell temperatures at **a** T_Cu_ = 840 °C; **b** T_Cu_ = 920 °C; **c** T_Cu_ = 990 °C; and **d** T_Cu_ = 1030 °C

Further confirmation of the composition of the nano-rods comes from the HRXRD results. Figure 3 displays the HRXRD profiles of samples grown using T_Cu_ = 860 and 1000 °C, showing that two extra peaks located at 27.4° and 64.5° are observed for the sample using T_Cu_ = 1000 °C. This indicates that some new phase(s) in the film was(were) formed at high Cu concentration, in fact the 2-theta values of both extra peaks match those of two reported diffraction peaks of hexagonal Cu-rich Cu-Te system [26]. It is worth pointing out that both TEM imaging and HRXRD results indicate that Cu-Te nano-rods are crystalline materials. As mentioned above, the Cu-Te nano-rods formed only near the surface region. This indicates that at high Cu concentration the incorporated Cu atoms prefer to migrate upward toward the surface of the thin film during growth. The results of a separated study using TOF-SIMS performed on several ZnTe:Cu samples grown using T_Cu_ = 840, 920, 990, and 1030 °C, as shown in Fig. 4a–d, support this claim. For sample grown using T_Cu_ = 840 °C, within the ZnTe:Cu layer, the concentration of CsCu + (black line) is nearly constant, which indicates that Cu atoms randomly substitute the Zn atoms and formed a uniform layer. For other three samples, Cu atoms are mainly distributed near the surface, becoming more obvious as T_Cu_ increases, which is consistent with the results obtained from TEM imaging and EDS analysis.

Having revealed the composition of the nano-rods, now we can address the unusual characteristics observed in the apparent resistivity data. It is well known that Cu-doped ZnTe thin films or bulk materials are p-type due to the substitutional incorporation of Cu atoms into the Zn lattice sites [24]. The observed initial rapid fall of the resistivity up to T_Cu_ = 870 °C is attributed to such a substitutional doping. As T_Cu_ further increases, the apparent resistivity experiences a more gradual decline up to T_Cu_ = 970 °C, which might be resulted from the appearing of saturation for the substitutional doping while the Cu-rich Cu-Te nano-rods with relatively low density may only lower down the apparent resistivity moderately. This is because even though Cu-rich Cu-Te alloy system has long been known as a highly degenerated p-type semiconductor [27], the ZnTe:Cu host lattice with relatively high resistivity is still dominated in size. However, further increase in T_Cu_ leads to a quantum leap in the transport mechanism. It is likely that for samples grown using T_Cu_ = 990 °C, the density of the Cu-Te nano-rods has reached a certain value and quantum tunneling for the hole carriers among neighboring nano-rods arises, leading to the observed sharp fall of the apparent resistivity.

**Fig. 5:**
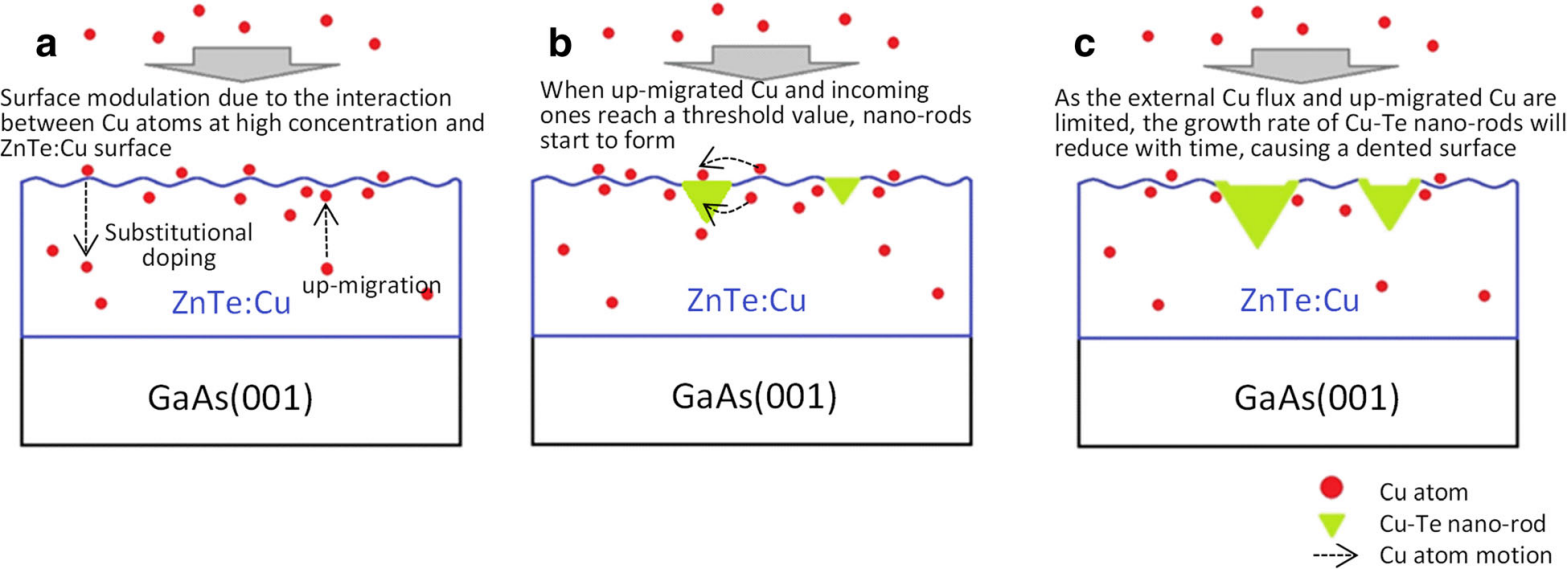
Schematic formation mechanism of 1D surface modulation and the Cu-Te nano-rods with **a**–**c** represent the progress order of the formation

Figure 5 addresses the formation mechanism and the geometrical shape of the Cu-Te nano-rods based on a phenomenological perspective. At high T_Cu_ condition, only part of the incoming Cu atoms will be incorporated as a substitutional dopant in the ZnTe lattice, the remaining Cu atoms would prefer to migrate upward toward the surface during the growth. When the as-grown thin film reaches a certain thickness, the concentration of the extra Cu atoms coming from the up-migration as well as from the source flux reaches a threshold value for the formation of Cu-rich Cu-Te nano-rods in some favorable sites. It is worth pointing out that the two embedded sides of the triangular cross-section of the nano-rods are found to be the members of the {111} plane family of ZnTe, which is based on the measured angle between these two sides and the surface normal of the sample, as shown in the insets of Fig. 2a, b. This observation is similar to the geometry of the ZnSe nanotrenches induced by mobile Au-alloy droplets as reported by us earlier [28]. Thus, it is expected that the formation of Cu-Te nano-rods shares some similarities with the formation of the ZnSe nanotrenches. The triangular cross-sectional shape of the Cu-Te nano-rods indicates that the growth of the Cu-Te nano-rods must proceed with a simultaneous dissociation of the neighboring as-grown ZnTe:Cu to expose the {111} planes that have low surface energy [29, 30]. This is well supported by the fact that the bonding strength of Cu-Te is 230.5 ± 14.6 kJ mol^–1^ while that of Zn-Te is 117.6 ± 18.0 kJ mol^–1^ [31]. The stronger bonding strength of Cu-Te over Zn-Te explains why the growth of Cu-Te nano-rods is more preferable accompanying with the dissociation of the neighboring ZnTe:Cu lattice when the concentration of extra Cu atoms is high enough. It is also worth mentioning that the triangular cross-sectional shape of the nano-rods also implies that the continuous growth of the nano-rods should require more and more Cu atoms. However, as the external Cu flux is fixed for a fixed Cu cell temperature and the Cu source from up-migration is also expected to be limited, one should expect that the growth of the nano-rods may proceed with reducing growth rate. In fact, this is likely to be the cause of their dented surfaces as seen by both the AFM and TEM images in Fig. 1e, f and Fig. 2b, respectively. Interestingly, as shown in Fig. 2b, the top edges near the dented surface of the nano-rods (indicated by solid arrows) are covered with a thin layer of Cu-Te thin film, which can be explained by the expected faster growth rate along the kink sites on the top edges since it is well known that a kink site has a lower energy than a site at the top surface of a nano-rod.

## Conclusions

In summary, we have carried out the MBE growth of a set of ZnTe:Cu thin films. It was found that 1D feature was observed in the RHEED patterns of thin films grown using T_Cu_ ≥ 870 °C, though this feature got dimmer with increasing Cu incorporation. The apparent resistivity of these films was observed to have an unusual sharp fall followed by a plateau for T_Cu_ ≥ 990 °C. Various post-growth characterizations including AFM, TEM, HRXRD, and SIMS reveal that at high Cu incorporation, substitutional doping reaches saturation and the remaining Cu atoms proceed with an up-migration. The accumulated Cu atoms at the surface, being supplied by both the up-migration and the source flux, modify the surface to form 1D surface modulation as well as forming Cu-Te nano-rods near the surface by dissociating their neighboring ZnTe:Cu lattice. We have provided a phenomenological model regarding the self-assembled formation mechanism of 1D surface modulation and nano-rods as well as illustrated how these nanostructures are related to the observed unusual RHEED patterns and apparent resistivity. Further studies on the exact Cu composition in the nano-rods as well as their crystal structure are underway and will be reported elsewhere.

## Additional file

Additional file 1: Supplementary material. (DOCX 1063 kb)

## Abbreviations

1D: One-dimensional
AFM: Atomic force microscopy
EDS: Dispersive X-ray spectroscopy
HRTEM: High resolution transmission electron microscopy
HRXRD: High resolution x-ray diffraction
RHEED: Reflection high energy electron diffraction
TOF-SIMS: Time-of-flight secondary ion mass spectrometry

## References

[1] Wang W, Lin AS, Phillips JD (2009). Intermediate-band photovoltaic solar cell based on ZnTe: O. Appl Phys Lett.

[2] Amin N, Sopian K, Konagai M (2007). Numerical modeling of CdS/CdTe and CdS/CdTe/ZnTe solar cells as a function of CdTe thickness. Sol Energy Mater Sol Cells.

[3] Wu Q, Hewitt TD, Zhang XC (1996). Two‐dimensional electro‐optic imaging of THz beams. Appl Phys Lett.

[4] Löffler T, Hahn T, Thomson M, Jacob F, Roskos H (2005). Large-area electro-optic ZnTe terahertz emitters. Opt Express.

[5] Wu Q, Litz M, Zhang XC (1996). Broadband detection capability of ZnTe electro‐optic field detectors. Appl Phys Lett.

[6] Boyer-Richard S, Robert C, Gérard L, Richters JP, André R, Bleuse J, Mariette H, Even J, Jancu JM (2012). Atomistic simulations of the optical absorption of type-II CdSe/ZnTe superlattices. Nanoscale Res Lett.

[7] Millerd JE, Brock NJ, Brown MS, DeBarber PA, Trivedi S (1996). Resonant holographic interferometry with ZnTe: V: Mn. Appl Optics.

[8] Kobayashi M, Mino N, Katagiri H, Kimura R, Konagai M, Takahashi K (1986). Growth of a ZnSe‐ZnTe strained‐layer superlattice on an InP substrate by molecular beam epitaxy. Appl Phys Lett.

[9] Li S, Jiang Y, Wu D, Wang L, Zhong H, Wu B, Lan X, Yu Y, Wang Z, Jie J (2010). Enhanced p-type conductivity of ZnTe nanoribbons by nitrogen doping. J Phys Chem C.

[10] Rouleau CM, Lowndes DH, McCamy JW, Budai JD, Poker DB, Geohegan DB, Puretzky AA, Zhu S (1995). Growth of highly doped p‐type ZnTe films by pulsed laser ablation in molecular nitrogen. Appl Phys Lett.

[11] Mondal A, Chaudhuri S, Pal AK (1989). Electrical properties of copper-doped ZnTe films. Thin Solid Films.

[12] Zhang L, Liu C, Yang Q, Cui L, Zeng Y (2015). Growth and characterization of highly nitrogen doped ZnTe films on GaAs (001) by molecular beam epitaxy. Mater Sci Semicond Process.

[13] Rakhshani AE, Thomas S (2013). Nitrogen doping of ZnTe for the preparation of ZnTe/ZnO light-emitting diode. J Mater Sci.

[14] Späth B, Fritsche J, Klein A, Jaegermann W (2007). Nitrogen doping of ZnTe and its influence on CdTe/ZnTe interfaces. Appl Phys Lett.

[15] Lee KS, Oh G, Kim EK (2015). Growth of p-type ZnTe thin films by using nitrogen doping during pulsed laser deposition. J Korean Phys Soc.

[16] Sakakibara S, Amano N, Ishino K, Ishida A, Fujiyasu H (1993). Characteristics of nitrogen-doped ZnTe films and ZnTe-ZnSe superlattices grown by hot wall epitaxy. Jpn J Appl Phys.

[17] Bohn RG, Tabory CN, Deak C, Shao M, Compaan AD, Reiter N (1994) RF sputtered films of Cu-doped and N-doped ZnTe. In Photovoltaic energy conversion, 1994., Conference record of the twenty fourth. IEEE Photovoltaic SpecialistsConference-1994, 1994 IEEE First World Conference, Vol. 1. IEEE, Piscataway, pp. 354–356

[18] Abazari M, Ahmad FR, Raghavan KC, Cournoyer JR, Her JH, Davis R, Chera J, Smentkowski V, Korevaar BA (2013) Carrier density in p-type ZnTe with nitrogen and copper doping. In MRS Proceedings, Vol. 1538. Cambridge University Press, Cambridge, pp. 383–389

[19] Aqili AK, Maqsood A, Ali Z (2001). Properties of copper-doped ZnTe thin films by immersion in Cu solution. Appl Surf Sci.

[20] Faulkner BR, Burst JM, Ohno TR, Perkins CL, To B, Gessert TA (2014) ZnTe: Cu film properties and their impact on CdS/CdTe devices. In 2014 IEEE 40th Photovoltaic Specialist Conference (PVSC). IEEE, Piscataway, p. 2321–2325

[21] El Akkad F, Thomas M (2005) Electrical and optical properties of rf co-sputtered ZnTe–Cu thin films. Physica Status Solidi (c) 1;2(3):1172–7.

[22] Jun Y, Kim KJ, Kim D (1999). Electrochemical synthesis of cu-doped znte films as back contacts to CdTe solar cells. Met Mater.

[23] Syed WA, Ahmed S, Saleem MS, Shah NA (2015). Cu-doped ZnTe thin films for potential energy applications. Chalcogenide Lett.

[24] El Akkad F, Abdulraheem Y (2013). Morphology, electrical, and optical properties of heavily doped ZnTe: Cu thin films. J Appl Phys.

[25] Wang G, Lok SK, Chan SK, Wang C, Wong GK, Sou IK (2009). The formation of an aligned 1D nanostructure on annealed Fe/ZnSe bilayers. Nanotechnology.

[26] Baranova RV, Avilov AS, Pinsker ZG (1974). Determination of the crystal structure of the hexagonal phase beta III in the Cu-Te system by electron diffraction. Soviet Physics, Crystallography.

[27] Zhu H, Luo J, Zhao H, Liang J (2015). Enhanced thermoelectric properties of p-type Ag2Te by Cu substitution. J Mater Chem A.

[28] Wang G, Lok SK, Sou IK (2011). ZnSe nanotrenches: formation mechanism and its role as a 1D template. Nanoscale Res Lett.

[29] Adachi S. Handbook on physical properties of semiconductors. Springer Science & Business Media; 2004. Kluwer Academic Publisher: Boston; 2004. Vol. 3, Chapter 9, p. 213.

[30] Meng Q, Jiang C, Mao SX (2008). Temperature-dependent growth of zinc-blende-structured ZnTe nanostructures. J Cryst Growth.

[31] Luo YR (2007) Comprehensive handbook of chemical bond energies. CRC press, Boca Raton

