# Supplementary material for: Self-Assembled Formation of Well-Aligned Cu-Te Nano-Rods on Heavily Cu-Doped ZnTe Thin Films
Source: Nanoscale Res Lett. 2016 Nov 29;11:531. doi: 10.1186/s11671-016-1741-x (PMC5126038; doi:10.1186/s11671-016-1741-x)
Supplement: Supplementary file 1 — Supplementary material. (DOCX 1063 kb) [file 11671_2016_1741_MOESM1_ESM.docx]

Self-assembled formation of well-aligned Cu-Te nano-rods on heavily Cu-doped ZnTe thin films

Jing Liang^1^, Man Kit Cheng^1^, Ying Hoi Lai^1^, Guanglu Wei^1^, Derman Yang Sean^1^, Gan Wang^2^, Sut Kam Ho^3^, Kam Weng Tam^3^ and Iam Keong Sou^1,^*

^1^Department of Physics and William Mong Institute of Nano Science and Technology, The Hong Kong University of Science and Technology, HKSAR, People’s Republic of China

^2^Department of Physics, South University of Science and Technology of China, 1088 Xueyuan Rd., Nanshan District, Shenzhen, Guangdong, People’s Republic of China

^3^Faculty of Science and Technology, University of Macau, E11 Avenida da Universidade, Taipa,

Macau, China

Supplementary Material

**I. TEM image and EDS characterization of a Cu-Te nano-rod**

A cross-sectional TEM image showing a triangular nano-rod of the sample which was grown using the Cu cell temperature at T_Cu_=1030 $℃$ is shown in FIG.S1(a), and two local positions, marked as 1 and 2, were examined by EDS analysis. FIG.S1(b) and (c) are EDS results in position 1 and 2, respectively. The corresponding quantitative chemical composition analysis is listed in the inserted tables. (The signals generated from Mo sample holder, surface oxidation and contaminants from sample preparation are not counted into calculation.) Outside the nano-rods, the chemical composition is mainly Cu-doped ZnTe with doping concentration around 5.5 at.%. While the composition of nano-rods is believed to be almost pure Cu-Te alloy with rich Cu concentration, though a small quantity of characteristic X-ray signal of Zn atoms was detected which is likely from the neighboring ZnTe:Cu layer. After subtracting the concentration percentage of Zn and Te signals from this layer, the Cu:Te ratio in nano-rods is close to 2:1, which is consistent with the HRXRD results that the two extra peaks in a sample grown using T_Cu_=1000$℃$ match the reported diffraction peaks of a hexagonal Cu-rich Cu-Te system [1].





**FIG. S1** (a)TEM image of the sample which was grown using the Cu cell temperature at T_Cu_ = 1030$\mathbf{℃}$; (b)EDS results in position 1; (c)EDS results in position 2. The corresponding quantitative chemical composition analysis is listed in the inserted tables.

**II. Why doesn’t the crystalline lattice of the Cu-Te nano-rods result in a distinctive feature in the RHEED patterns of ZnTe:Cu thin films grown at high T_Cu_?**

Ewald construction tells us that the observed RHEED patterns of a sample surface are the interceptions of the Ewald sphere with the reciprocal lattice of the sample surface [2]. As addressed in our previous report [3], for a [110]-oriented 1D surface structure with uneven groove spacing, its corresponding reciprocal lattice is a plane perpendicular to both the grooved surface and the orientation of the grooves. However, for the aligned 1D surface structure observed for the either the ZnSe surface [4] or the ZnTe surface described in our current study, the lattice spacing is still maintained along the 1D grooves, one should expect that its corresponding reciprocal lattice should consist of a group of planes with a spacing corresponding to the reciprocal lattice spacing of the host lattice along the [110] direction. As mentioned in the main text of this paper, for those ZnTe:Cu thin films with high Cu temperature, the surface contains dented 1D surface structure contributed by the Cu-Te nano-rods that are crystalline materials as illustrated by both our cross-sectional TEM and HRXRD studies. One may ask why the crystalline lattice of the Cu-Te nano-rods do not result in a distinctive feature in the background 1D RHEED patterns attributed to the surface modulation. The answer of this question comes from the fact that the 1D surface structure has removed the periodicity in two directions, one is along the surface normal and the other is perpendicular to the 1D grooves. Only the periodicity along the 1D grooves is preserved. FIG.S2 displays the high-resolution plane-view TEM image of a ZnTe:Cu sample grown using T_Cu_ =1030$℃$, where the ZnTe:Cu region and the Cu-Te nano-rod region are specified with their corresponding Fourier transform patterns shown on the right side. Inspection of these Fourier transform patterns tells that the crystalline lattice of the Cu-Te nano-rod lattice has a lateral misalignment of ~5.5 degrees off the ZnTe:Cu lattice (angle between the two white lines shown in FIG.S2(c)). Since the Fourier transform patterns are equivalent to the reciprocal lattice, this lateral misalignment implies that reciprocal lattice periodicity of the Cu-Te nano-rod along the [110] direction will be around 11.6 times larger than the corresponding lattice periodicity of the ZnTe:Cu surface. With this understanding, one should expect that the reciprocal plane spacing of the Cu-Te nano-rods is too large and its corresponding RHEED streak spacing, even in their narrowest case (when the e-beam is along the [$1\bar{1}0$] direction), will still be too large to be observed in the RHEED screen. Thus, it explains that the role of the Cu-Te nano-rods on the RHEED observation will only lead to a dimmer 1D RHEED pattern without giving out any distinctive feature associated with their crystalline periodicity.





**FIG. S2** (a)High resolution Plan-view TEM image of a ZnTe:Cu sample grown using T_Cu_=1030 $℃$. Corresponding Fast Fourier transform (FFT) patterns of (b) ZnTe:Cu and (c)Cu-Te nano-rod lattice.

**III. Further discussion about the resistivity plateau region for T_Cu_**$\boldsymbol{\geq}$**990**$\mathbf{℃}$

The observation of a plateau region in the apparent resistivity does not really imply that the resistivity of the overall Cu-Te nano-rods remains the same as T_Cu_ increases, instead, it actually increases as T_Cu_ increases. The reason behind this argument is that the apparent resistivity, ρ, presented in Fig.1(c) is derived using the product of the measured sheet resistance, R_s_, and the thickness of the thin film, t (i.e.,$\rho=R_{s}\times t$), with the latter assumed to be the same for the whole set of ZnTe:Cu thin films. The apparent resistivity values can approximately represent the true values only for those samples with negligible contribution from Cu-Te nano-rods. However, for samples grown using T_Cu_≥990 $℃$, the size of the Cu-Te nano-rods actually increases with T_Cu_. The observation that the apparent resistivity data of these samples form a plateau only indicates these samples have very close sheet resistance but their true resistivity increases with T_Cu_. This can be understood if the over-doped condition is reached for T_Cu_ ≥ 1000 $℃$. Over-doped status is in fact quite common for many doped semiconductors [5-7]. A possible reason behind this over-doped status is that as Cu cell temperature increases to a certain value, Cu interstitial and/or Te vacancy defects, which have an n-type character, are generated to compensate the p-type character of Cu vacancy defects in Cu-Te nano-rods, thus increasing the resistivity of the nano-rods.

REFERENCES

1. Baranova RV, Avilov AS, Pinsker ZG. Determination of the Crystal Structure of the Hexagonal Phase Beta III in the Cu-Te System by Electron Diffraction. Sov. Phys. Crystallogr. 1974 May;18(6):736-40.
2. Kawamura T, Hasebe M, J. Dobson P. The Origin of Circular Arc in RHEED: 1D Ordered Surface. Journal of the Physical Society of Japan. 1985 Oct;54(10):3675-8.
3. Wang G, Lok SK, Chan SK, Wang C, Wong GK, Sou IK. The formation of an aligned 1D nanostructure on annealed Fe/ZnSe bilayers. Nanotechnology. 2009 May 6;20(21):215607.
4. Wang G, Lok SK, Sou IK. ZnSe nanotrenches: formation mechanism and its role as a 1D template. Nanoscale research letters. 2011 Mar 30;6(1):1.
5. Shukla RK, Srivastava A, Srivastava A, Dubey KC. Growth of transparent conducting nanocrystalline Al doped ZnO thin films by pulsed laser deposition. Journal of crystal growth. 2006 Sep 4;294(2):427-31.
6. Kim H, Gilmore CM, Pique A, Horwitz JS, Mattoussi H, Murata H, Kafafi ZH, Chrisey DB. Electrical, optical, and structural properties of indium–tin–oxide thin films for organic light-emitting devices. Journal of Applied Physics. 1999 Dec 1;86(11):6451-61.
7. Craciun MF, Rogge S, den Boer MJ, Margadonna S, Prassides K, Iwasa Y, Morpurgo AF. Electronic Transport through Electron‐Doped Metal Phthalocyanine Materials. Advanced Materials. 2006 Feb 3;18(3):320-4.
